# Susceptibility of Beavers to Chronic Wasting Disease

**DOI:** 10.3390/biology11050667

**Published:** 2022-04-26

**Authors:** Allen Herbst, Serene Wohlgemuth, Jing Yang, Andrew R. Castle, Diana Martinez Moreno, Alicia Otero, Judd M. Aiken, David Westaway, Debbie McKenzie

**Affiliations:** 1 U.S. Geological Survey National Wildlife Health Center, Madison, WI 53711, USA; aherbst@usgs.gov; 2 Centre for Prions and Protein Folding Diseases, University of Alberta, Edmonton, AB T6G 2R3, Canada; slw1@ualberta.ca (S.W.); jyang6@ualberta.ca (J.Y.); acastle@ualberta.ca (A.R.C.); dam3@ualberta.ca (D.M.M.); judd.aiken@ualberta.ca (J.M.A.); david.westaway@ualberta.ca (D.W.); 3 Department of Agricultural, Food and Nutritional Sciences, University of Alberta, Edmonton, AB T6G 2R3, Canada; 4 Department of Medicine, University of Alberta, Edmonton, AB T6G 2R3, Canada; 5 Department of Biological Sciences, University of Alberta, Edmonton, AB T6G 2R3, Canada; 6 Centro de Encefalopatias y Enfermedades Transmisibles Emergentes, University of Zaragoza, 50009 Zaragoza, Spain; aliciaogar@unizar.es

**Keywords:** prions, chronic wasting disease, beavers, wildlife diseases

## Abstract

**Simple Summary:**

Chronic wasting disease is increasing across the landscape, and this is threatening other wildlife species in addition to cervids. Our objective was to evaluate the possibility that chronic wasting disease could transmit to beavers. Our results indicate that beavers are susceptible to multiple types of prion diseases, including chronic wasting disease.

**Abstract:**

Chronic wasting disease (CWD) is a contagious, fatal, neurodegenerative prion disease of cervids. The expanding geographical range and rising prevalence of CWD are increasing the risk of pathogen transfer and spillover of CWD to non-cervid sympatric species. As beavers have close contact with environmental and food sources of CWD infectivity, we hypothesized that they may be susceptible to CWD prions. We evaluated the susceptibility of beavers to prion diseases by challenging transgenic mice expressing beaver prion protein (tgBeaver) with five strains of CWD, four isolates of rodent-adapted prions and one strain of Creutzfeldt–Jakob disease. All CWD strains transmitted to the tgBeaver mice, with attack rates highest from moose CWD and the 116AG and H95+ strains of deer CWD. Mouse-, rat-, and especially hamster-adapted prions were also transmitted with complete attack rates and short incubation periods. We conclude that the beaver prion protein is an excellent substrate for sustaining prion replication and that beavers are at risk for CWD pathogen transfer and spillover.

## 1. Introduction

Chronic wasting disease (CWD) is a contagious, fatal, neurodegenerative disease of cervids first identified in 1967 [1]. CWD has spread within the United States of America to 29 states and within Canada to four provinces [2]. CWD has been detected in captive cervids in Korea [3], and more recently in wild reindeer [4], moose [5] and red deer [6] in Northern Europe. CWD prevalence increases with time, with some enzootic areas approaching rates of 50%, depending upon cervid density and habitat [7].

CWD, like all transmissible spongiform encephalopathies, is caused by the template-dependent conversion of the normal host-encoded cellular prion protein, PrP^C^, to an abnormal disease-specific prion protein isoform, PrP^CWD^ [8]. The primary structure of the host PrP^C^ and the tertiary and quaternary structures of the invading PrP^CWD^ are the most important factors for interspecies transmission. A single amino acid change in the PrP^C^ or alteration in the conformation (strains) of the invading PrP^CWD^ is sufficient for CWD resistance or death of the host [9,10]. Different strains of CWD show distinct phenotypes (biochemical properties, incubation period, neurotropism, and host range) and stable manifestation of those properties over passage (inheritance). In many cases, prion biologists have reconstructed the phylogenetic relationships between distinct prion strains [11,12,13] including in CWD [9]. At least six strains of CWD have been identified.

Unlike many other prion diseases, CWD is contagious and infectivity has been measured in saliva, urine, feces and antler velvet [14,15,16,17,18] from infected cervids. Shed CWD prions persist in the environment for years [19,20,21,22] and the infectivity is a substantial force that maintains the CWD epizootic. The presence of shed CWD infectivity has important implications for exposed sympatric species that are at risk for pathogen transfer events. CWD prions have been shown to be experimentally transmissible to multiple non-cervid sympatric species including cattle [23], sheep [24,25], pigs [26], cats [27], racoons [28,29], bank voles [30], meadow voles, red-backed voles, white-footed mice and deer mice [31].

Among the many species at risk of CWD pathogen transfer, beavers have unique exposures to CWD infectivity that arise from their role as ecosystem engineers. Beavers are keystone species that improve habitats and increase species diversity. Two species of beavers (*Castor canadensis* and *Castor fiber*) are sympatric with the CWD epidemics in North America and Europe. Both species share the same prion protein primary sequence, which contains six octapeptide repeats as determined from analysis of sequencing data (PRJNA313146) and confirmed by Sanger sequencing (data not shown). Homology (Figures S1 and S2) is strong between the prion protein of beavers, other rodents and cervids. Beavers create, maintain and extend riparian habitats by impounding water and opening up the tree canopy. These activities improve cervid habitat by maintaining water on the land and enhancing plant growth; high densities of deer are associated with riparian habitats [32]. In riparian areas, the shedding of prions by infected cervids may expose beavers to CWD prions. Polydipsia (excessive thirst) is a clinical symptom of CWD and this would concentrate shedding in riparian areas [33]. Beavers dig and carry mud to build dams and lodges and the binding of prions to soil and clays enhances oral infectivity [20,21]. Beavers eat, cut, carry and store tree bark that may have been used by deer as a rub to remove infectious velvet. Beavers and cervids are herbivores and they compete for food sources including grasses, willow and aspen [34]. Grasses bind prions, increasing CWD bioavailability [35]. These ecological and physical interactions between beavers and cervids indicate that beavers may be exposed to CWD and, thus, at risk for infection.

To evaluate the susceptibility of beavers to prion disease, we generated transgenic mice expressing the beaver prion protein and challenged them with various CWD and rodent prion strains. We found that there is a low molecular species barrier to conversion of beaver PrP by multiple prion strains including strains of CWD.

## 2. Materials and Methods

All work with animals was performed in compliance with the Canadian Council on Animal Care Guidelines and Policies. All procedures involving animals were reviewed and approved by the Health Sciences Animal Care and Use Committee of the University of Alberta.

### 2.1. Creation of Beaver Prnp Transgenic Mice (tgBeaver)

We graciously received *Castor canadensis* and *Castor fiber* tissues collected by Bill Abercrombie or Bozena Szafranska, respectively. Muscle from *Castor canadensis* was powdered with a mortar and pestle under liquid nitrogen and the DNA extracted from 50 mg of tissue using proteinase K digestion, phenol/chloroform extraction and ethanol precipitation. The beaver *Prnp* open reading frame (ORF) was amplified by polymerase chain reaction (PCR) using the GoTaq Long PCR Master Mix (Promega Inc., Madison, WI, USA) and primer set 1 (Table S1). The PCR product was gel purified and cloned into the PGEM T-Easy vector (Promega). Clones were verified by Sanger sequencing [36]. Plasmid DNA containing the beaver *Prnp* ORF was used as the template for subsequent PCR reactions with primer set 2 (Table S1). The resulting amplicon was again cloned into PGEM T-easy and the clone sequence verified by Sanger sequencing. XhoI and MluI digestion were used to separately prepare an insert containing the *Prnp* ORF and the MoPrP.*Xho*.Mod ‘half-genomic’ transgenesis vector [37,38,39]. A clone containing the 15.27 Kb construct was verified by restriction digestion mapping and sequencing.

Plasmid DNA for pronuclear injection was purified using the EndoFree Plasmid Maxi Kit (Qiagen, Redwood City, CA, USA) and digested with *Not*I. The digested product was electrophoresed on a 0.7% agarose gel. Lanes containing ladder and 1 lane of digested DNA were separated and stained with SYBR Safe DNA Gel Stain (Thermo Fisher Scientific, Waltham, MA, USA). The region of the gel corresponding to the transgene was cut out and purified with UltraClean GelSpin DNA Extraction Kit (Mo Bio Laboratories, Inc., Carlsbad, CA, USA) and eluted in EmbryoMax Injection Buffer (Millipore, Burlington, VT, USA). DNA concentration was determined and diluted to 1.5 pg/μL immediately prior to injection. Pronuclear microinjections were performed at the University of Alberta’s Transgenic Core Facility. Briefly, superovulated FVB *Prnp*-knockout females were mated with males of the same genotype. Zygotes were collected at 0.5 days post coitus and a single pronuclei was microinjected with DNA at a 1.5 pg/μL concentration using a XenoWorks digital microinjector (Sutter Instrument, Novato, CA, USA). Viable zygotes were surgically transplanted into the oviducts of pseudopregnant CD1 females (Charles River Laboratories, Wilmington, NC, USA).

Four lines of founder transgenic mice were evaluated by capillary Western assay for expression of beaver PrP. Mice were euthanized by cervical dislocation. Tissue was immediately extracted and frozen on dry ice. Using a Dounce homogenizer, hemisected brain samples were manually homogenized in PBS (Boston Bioproducts, Ashland, OR, USA) containing protease inhibitor cocktail (Roche, Indianapolis, IN, USA) and 1 mM EDTA. Total protein concentrations were determined by bicinchoninic acid assay (Thermo Scientific, Waltham, MA, USA).

Assays were prepared using a 12–230 kDa Separation Module and Anti-Mouse and Anti-Rabbit Detection Modules (ProteinSimple; SM-W004, DM-002 and DM-001, respectively, Biotechne Inc., San Jose, CA, USA). All reagents used were from these kits unless stated otherwise. Brain homogenate samples were denatured and reduced by incubating at 95 °C for 5 min in the presence of Fluorescent Master Mix. Samples, blocking buffer, primary and secondary antibodies, chemiluminescence reagents and wash buffer were loaded according to the manufacturer’s instructions into microtitre plates pre-filled with proprietary electrophoresis buffers. Primary antibodies were Sha31 anti-PrP (1:10,000; Bertin Bioreagent, Rockville, MD, USA) and anti-β-tubulin (1:200; Biotechne Inc., San Jose, CA, USA). Plates and capillaries were loaded into a Wes capillary Western instrument [40] (Biotechne Inc., San Jose, CA, USA) and assays were performed using the default 12–230 kDa separation range protocol with the separation time adjusted to 30 min. Artificial lane view images were created from the original chemiluminescent spectra using Compass for SW, version 6.0.0 (ProteinSimple Inc., San Jose, CA, USA), with visual adjustment performed using the built-in contrast slider. The increased apparent molecular weight of PrP is a consistent, systematic error for this technique and was addressed in-depth in an earlier publication [41].

### 2.2. Bioassay of CWD and Other Prions in tgBeaver Mice

Cohorts of male and female weanling tgBeaver mice were intracerebrally inoculated via the anterior fontanelle with 30 μL of a 10% *w/v* brain homogenate aseptically prepared in sterile water. Brain tissues used to prepare homogenates were collected from cervids clinically affected with CWD (Wisc-1 [9], H95+ [9], CWD2 from elk [42,43]), cervidized transgenic mice (tg60 Moose [5], tg60 116AG [44], tg33 Wisc-1 [9], tg60 H95+ [9]), or other rodent-adapted prions (Chandler/RML [45,46], rat-adapted scrapie (RAS) [47], Hyper (Hy) [48,49], Drowsy (Dy) [48,49]). The sporadic Creutzfeldt–Jakob disease (CJD) prions used in these experiments were patient samples passaged in 129 M tgHuman mice [50] received from Robert Rohwer. The cervidized transgenic mice lines tg33 and tg60 express alleles of the white-tailed deer prion protein that differ at amino acid 96. Tg33 encode glycine at residue 96 whereas tg60 mice encode serine. These lines express similar amounts of PrP^C^ as deer [51]. Mice were euthanized by CO_2_ asphyxiation throughout the study for intercurrent conditions and/or neurodegeneration consistent with a clinical onset of prion disease. Incubation periods are the mean +/− standard error. The experiment was terminated at 647 days post infection. Positive transmission of prions was determined by western blot analysis of proteinase K (PK) treated brain homogenates and/or immunohistochemical deposition of prion protein.

Mouse brain tissues were homogenized to 10% (*w/v*) in sterile water with a tissue disruptor (Omniprep) and disposable homogenization tubes and beads. Brain homogenates were analyzed for proteinase K-resistant prion protein (50 μg/mL, Roche) by enzymatic digestion and Western blotting as described [10]. Western blots were probed with the monoclonal antibody Bar224 (1:10,000, Cayman Chemical, Ann Arbor, MI, USA) [52] or 12B2 (1:10,000, Wageningen Bioveterinary Research, Lelystad, NL) [53]. In some cases, brain tissue was collected for histological analysis. Sagittal hemispheres were fixed in formalin prior to paraffin embedding. Sagittal sections were cut and stained with hematoxylin and eosin or immunostained using Bar224 for PrP deposition as described in [9].

## 3. Results

**Figure 1:**
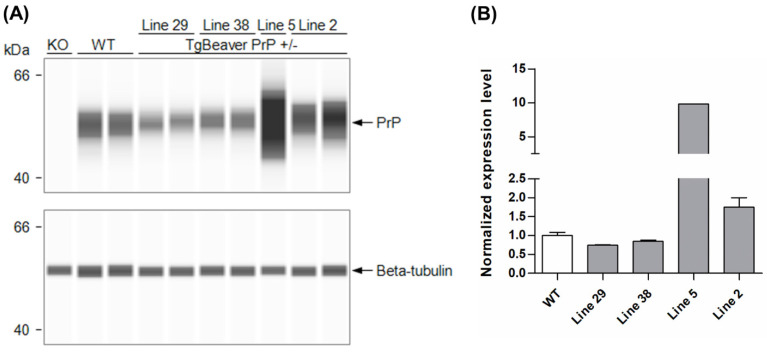
Generation and evaluation of transgenic mice expressing beaver *Prnp*. (**A**) Capillary Western images and (**B**) chart showing PrP^C^ expression levels (mean values ± S.D.) in brain homogenates of hemizygous TgBeaver PrP mice derived from several different transgenic founders. PrP^C^ signals were corrected for loading error based on beta-tubulin signals before normalization against the WT mean value. The TgBeaver mice were homozygous null for the endogenous mouse *Prnp* locus. The monoclonal antibody Sha31 was used to detect PrP^C^. KO, knockout; WT wild-type FVB mice.

Standard pronuclear microinjection procedures were followed to introduce the tgBeaver PrP expression construct into FVB *Prnp*-knockout zygotes. The founders identified were bred with FVB *Prnp*-knockout mice, and the hemizygous transgenic F1 progeny screened for PrP^C^ expression using a capillary Western assay. Of the four transgenic lines tested, line 2 was chosen for use in prion infection experiments. PrP^C^ expression in the brain was 1.76× that of wild-type mice (Figure 1).

Cohorts of tgBeaver mice (line 2) were challenged with five strains of CWD, four isolates of rodent-adapted prions and one strain of CJD to test the capacity of beaver PrP^C^ to sustain prion replication and induce prion disease (Table 1, Figures S3 and S4). Fulminant prion disease with complete attack rates and short incubation periods were observed when the tgBeaver mice were infected with the rodent prions. The Hy strain of transmissible mink encephalopathy was particularly virulent. CJD prions did not observably replicate in the tgBeaver mice. Among the CWD strains, the H95+ and 116AG prions showed the highest attack rates. By comparison, the Wisc-1 strain transmitted poorly. CWD strains that had been passaged in transgenic cervidized mice had shorter incubation periods and higher attack rates than isolates from deer. A sex difference was indicated, with higher attack rates and/or shorter incubation periods in the male tgBeaver mice. Cox proportional hazards regression analysis was performed using Prism 9.3 (Graphpad Inc., San Diego, CA, USA). The hazard estimate for male sex was 2.36 as compared to female sex with a 95% confidence interval of 1.388 to 4.029. A model comparison between sex and prion agent versus prion agent alone preferred the sex and agent model, (*p* = 0.0016).

**Figure 2:**
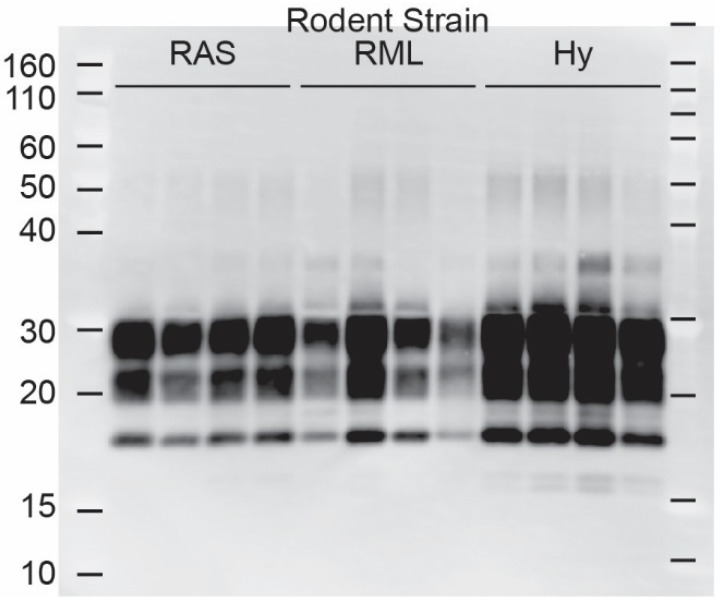
TgBeaver-adapted rodent prions. TgBeaver mice were inoculated with rodent-adapted scrapie from rats (RAS), or mice (RML) or hamster-adapted transmissible mink encephalopathy (Hy). 1 The 0% brain homogenates were digested with 50 μg/mL of PK and analyzed by Western blotting. The monoclonal antibody Bar224 was used to detect beaver PrP-res at a dilution of 1:10,000.

**Figure 3:**
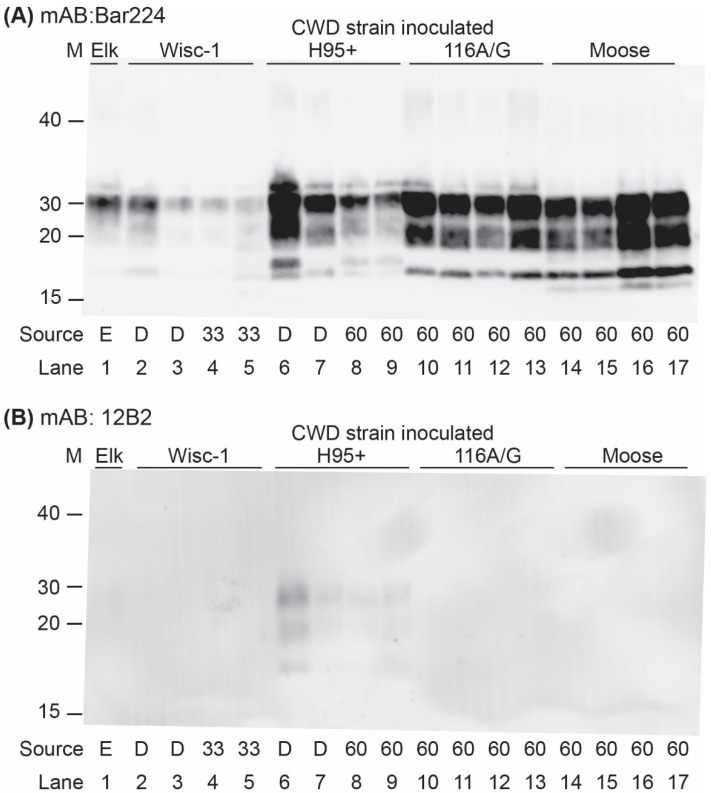
TgBeaver-adapted CWD prions. TgBeaver mice were inoculated with CWD prions from Elk (lane 1; E), white-tailed deer (lanes 2, 3, 6, 7; D), tg33 96G cervidized mice (lanes 4, 5; 33), or tg60 96S cervidized mice (all other lanes; 60). The 10% brain homogenates were digested with 50 μg/mL of PK and analyzed by Western blotting. (**A**) The monoclonal antibody Bar224 (1:12,500) was used to detect beaver prions. (**B**) The N-terminal binding antibody 12B2 (1:3333) only recognizes the beaver-adapted H95+ CWD prions.

Western blot analysis of brain homogenates from the infected tgBeaver mice identified multiple strain-dependent PrP-res types (Figure 2 and Figure 3). Only H95+ containing prions were recognized by the 12B2 antibody. 12B2 recognizes the N-terminal portion of the prion protein and this region was protected from proteinase K digestion in the tgBeaver-adapted H95+ prions. In contrast to the rodent prions (RML, Hy and RAS), the proteinase K-resistant PrP from the CWD strains showed several distinct patterns, consistent with strain-specific transmission. Uniform strain-specific banding patterns were typically observed. In the case of the H95+ deer prions, however, two banding patterns were observed (Figure 3, lanes 6 and 7). The H95+ deer isolate used for the transmission studies is composed of a mixture containing a majority of H95+ prions with a minor component of Wisc-1 prions, while the H95+ from the tg60 mice is highly enriched in the H95+ prions [9]. The divergent patterns in lanes 6 and 7 likely arose by selection in the tgBeaver mice, the H95+ isolate selected in the lane 6 sample, and the Wisc-1 isolate selected in the lane 7 sample. In some samples (lanes 6, 8, 9, and 13–17), an extra band was observed either above or below the un-glycosylated band. This doublet was strain specific.

**Figure 4:**
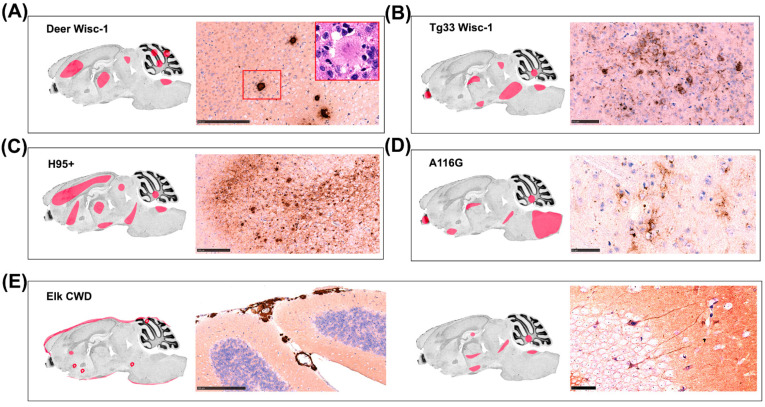
Distribution and variety of neuropathological changes observed in tgBeaver mice inoculated with several CWD strains. Regions with extensive neuropathological change are highlighted in pink. (**A**) PrPd plaques were observed by immunohistochemical staining in mice inoculated with deer Wisc-1. Inset picture (hematoxylin & eosin staining) shows the characteristic morphology of a florid plaque, surrounded by a halo of spongiform degeneration. (**B**) Intraneuronal PrPd aggregates were abundant in mice inoculated with tg33 Wisc-1 prions. (**C**) Abundant and widely distributed plaques and granular deposits were observed in one mouse inoculated with H95+ strain. (**D**) Stellate PrPd deposits, associated with reactive glia, were detected in mice challenged with the A166G strain. (**E**) Subpial and perivascular deposits were observed in one mouse inoculated with elk CWD2 strain (**left**), whereas other mice presented with intraneuronal and axonal deposits (**right**). Immunohistochemical staining for PrP was performed using mAB Bar224 1:1000. Scale bar is 200 μm for panels (**A**,**E**) (**left**) and 50 μm for panels (**B**–**E**) (**right**).

Brain samples from some of the tgBeaver mice were analyzed histopathologically and immunohistochemically. The different CWD strains resulted in a variety of morphological and distribution patterns of disease-related PrP (PrPd) deposition in the brains of tgBeaver mice, as expected on first interspecies passage. Florid plaques were observed in the tgBeaver mice inoculated with deer-derived Wisc-1, whereas tg33 mouse-passaged Wisc-1 produced smaller, intraneuronal deposits. PrPd plaques and intraneuronal deposits were also observed in some mice inoculated with the H95+ prions. Stellate deposits affecting reactive glia were abundant in animals inoculated with 116AG. Elk prions produced abundant subpial and perivascular PrPd aggregates in some animals and more discrete intracellular accumulation in others (Figure 4, Figure S5).

## 4. Discussion

Transgenic mice expressing beaver PrP^C^ are susceptible to multiple prion strains, after intracranial challenge, including those that cause CWD. Strain-specific attack rates and incubation periods were observed in the tgBeaver mice, with the H95+ strain showing the highest virulence among the CWD strains tested. Transmission of the CWD isolates was associated with protracted and variable incubation periods and variable attack rates, as is typical for first passage interspecies transmission of prions. No correlation was apparent in the degree of homology between PrP^C^ in the donor and recipient animals and transmission. Beaver PrP^C^ has the highest homology to human PrP^C^ (92.5%), as compared to the rodents, yet the human CJD prions obtained by passage in tgHuman mice did not result in clinical or subclinical infections. By contrast, the rodent prions had the highest attack rates and shortest incubation periods, even though the sequence homology was the lowest (Figure S2). With the exception of CJD transmission, higher attack rates and shorter incubation periods were observed when the source prion strain was derived from rodents. For example, Wisc-1 prions from deer transmitted poorly compared to the same prion strain passaged in the tg33 transgenic cervid mice. Rodents typically accumulate very high titres of prion infectivity per gram of brain tissue and the higher concentration of prions in the inocula from the cervidized mice may have facilitated transmission.

After the prion inoculation experiments were initiated, we noted that all transgene-positive offspring of crosses between transgene-positive males and *Prnp*-knockout females were female, indicating that the tgBeaver PrP expression transgene had integrated into the X-chromosome. While sex-specific differences in prion transmissions are per se thought to be minor [54], due to X-inactivation, it is possible that the female transgenic mice had lower PrP^C^ expression levels than the male transgenics. This could explain the tendency for female mice to display longer and more variable incubation periods for most (although not all) of the prion agents tested.

Intracranial infection is a gold-standard laboratory method to measure the molecular barrier between a recipient PrP^C^ and an invading prion strain; however, this approach alone cannot determine the risk of pathogen transfer, which also requires exposure. CWD is geographically expanding, increasing its prevalence within endemic regions, and increasing CWD prion environmental contamination [55]. These factors are increasing the exposure of all sympatric species to CWD prions, including beavers, and driving the risk of interspecies transmission of CWD. High levels of exposure can override even strong species barriers, as was shown by the transmission of bovine spongiform encephalopathy to humans. CWD prion transfer to beavers may result in pathogen spillover facilitated by long beaver lifespans (>20 years) that would enable CWD adaptation and shedding and social interactions that would facilitate transmission.

Transmission studies demonstrate that CWD prions can infect a variety of other species including cattle, sheep, pigs, ferrets, hamsters, red-backed and bank voles, and white-footed mice. Our data indicate that beavers also are expected to be susceptible to CWD and at risk for pathogen transfer and spillover. We are unaware of any attempt to perform surveillance for prion disease in wild beavers. Targeted or opportunistic surveillance of wild beavers in areas where CWD, in cervids, has reached high prevalence would further clarify the risk of CWD to beavers.

## Figures and Tables

**Table 1:** Attack rate and incubation periods in the transgenic beaver mice.

Inoculum (Strain, Source)	Attack Rate		Incubation Period (mean ± SD)	
CWD	Male	Female	Male	Female
Wisc1, Deer	1/4 *	1/4 *	-	-
Wisc1, tg33	4/6	4/6	333.3 ± 60.6	355.3 ± 76.7
H95+, Deer	4/4	6/7	388.5 ± 109.1	373.8 ± 83.5
H95+, tg60	6/6	4/6	204.8 ± 24.8	291.3 ± 68.0
CWD2, Elk	1/5 *	3/5 *	-	-
116AG, tg60	3/6	7/7	398.3 ± 44.4	518.3 ± 37.6
Moose, tg60	3/5	2/7	215.3 ± 24.0	497.5 ± 50.2
Other prions				
CJD, tgHuman	0/4	0/1	-	-
RML, mouse	1/1	4/4	122	260.0 ± 86.8
Hy, hamster	3/3	7/7	76.7 ± 16.3	115.9 ± 15.3
Dy, hamster	0/0	2/7 *	-	-
RAS, rat	7/7	3/3	101.3 ± 1.5	139.1 ± 34.0

* Subclinical infections were identified in some non-clinical mice euthanized at the end of the experiment by Western blotting for proteinase K-resistant prion protein. See Materials and Methods for sources of prion isolates.

## Data Availability

The data presented in this study are contained within this article and Supplementary Material.

## Supplementary Materials

The following supporting information can be downloaded at: https://www.mdpi.com/article/10.3390/biology11050667/s1, Figure S1: sequence alignment of prion proteins.; Figure S2: Homology of prion protein primary structure between beavers, cervids, other rodents and humans; Figure S3: Survival Curves of tgBeaver mice challenged with indicated prion strains; Figure S4: Dot plots of incubation periods in tgBeaver mice; Figure S5: Specificity of immunohistochemical staining in tgBeaver mice; Table S1: PCR primers.
