# Supplementary material for: Susceptibility of Beavers to Chronic Wasting Disease
Source: Biology (Basel). 2022 Apr 26;11(5):667. doi: 10.3390/biology11050667 (PMC9137852; doi:10.3390/biology11050667)
Supplement: Supplementary file 1 [file biology-11-00667-s001.zip › biology-1664946-supplementary.pdf]

Supplemental Materials

Table S1. PCR primers

Primer Set	Forward	Reverse
one	5′- ATC TGT CAT GGC GAA CCT TG -3′	5′- GGG ACA AAG AGA GAA GCA AGA A -3′
two	5′- ATC TCG AGA GTC CAA TTT AGG AGA GCC AAG CAG ACT ATA AGT CAT CAT GGC GAA CCT TGG CTG CTG GCT GC -3′	5′- TGA CGC GTT CAT CCC ACT ATC AGG AAG ATG AGG -3′

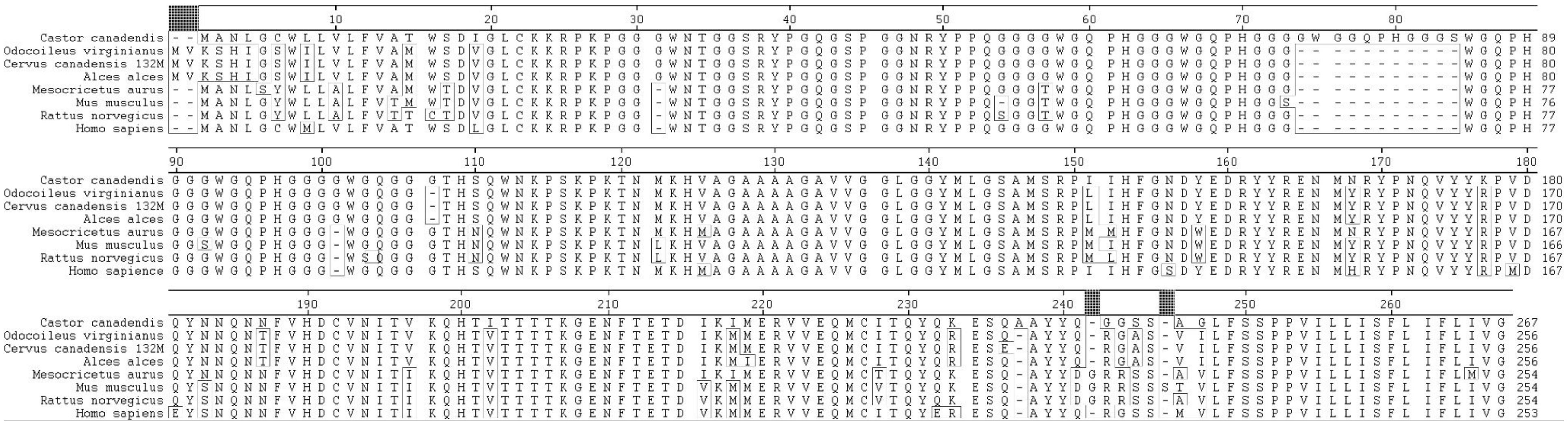

Figure S1. Sequence alignment of prion proteins.

Percent Identity

Divergence	1	2	3	4	5	6	7	8		
1		91.4	91.0	91.0	90.6	89.4	88.6	92.5	1	Castor canadendis
2	7.9		99.6	99.6	87.4	86.6	86.6	89.7	2	Odocoileus virginianus
3	8.3	0.4		99.2	87.0	86.2	86.2	89.3	3	Cervus canadensis 132M
4	8.3	0.4	0.8		87.0	86.2	86.2	89.3	4	Alces alces
5	8.8	11.6	12.1	12.1		94.1	94.1	89.7	5	Mesocricetus aurus
6	11.1	11.6	12.1	12.1	5.8		96.5	89.7	6	Mus musculus
7	11.1	13.0	13.5	13.5	6.2	2.8		89.7	7	Rattus norvegicus
8	7.5	9.3	9.8	9.8	11.1	10.7	11.1		8	Homo sapiens
	1	2	3	4	5	6	7	8		

Figure S2. Homology of prion protein primary structure between beavers, cervids, other rodents, and humans.

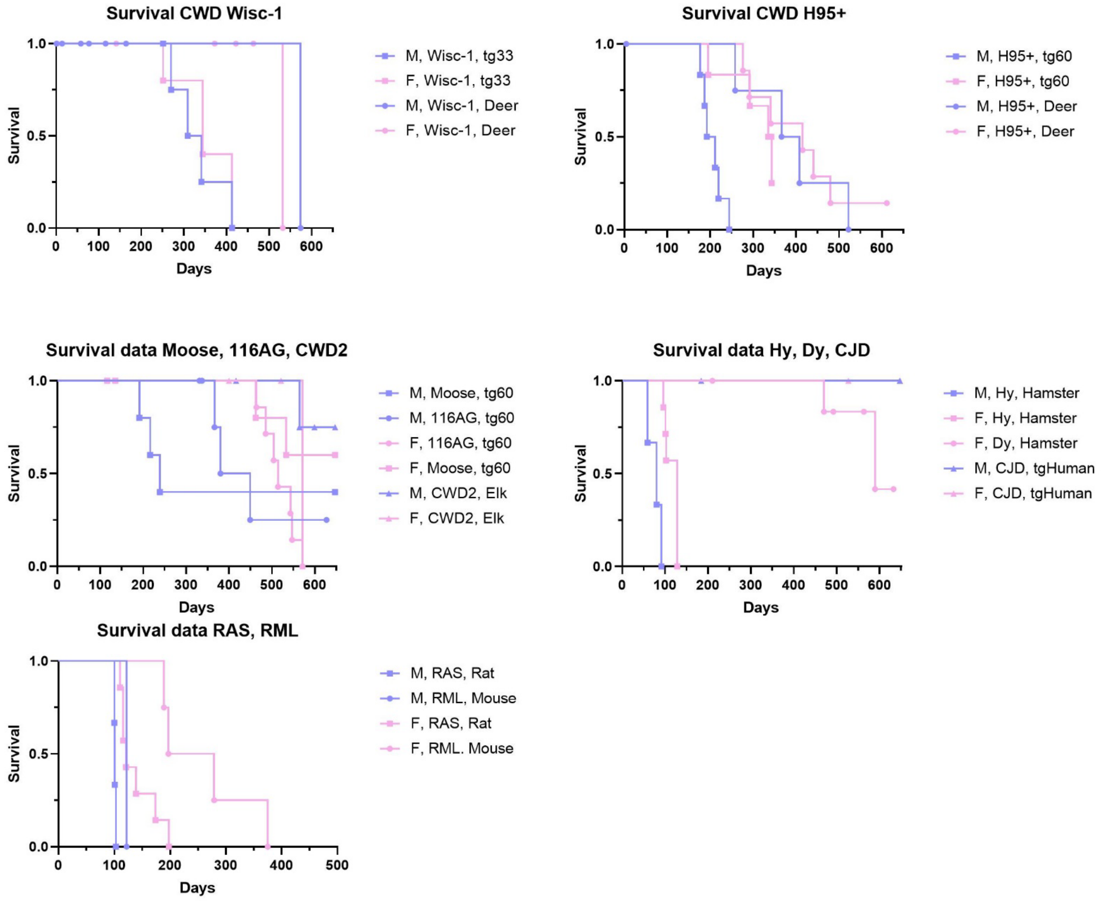

Figure S3. Survival curves of tgBeaver mice challenged with indicated prion strains.

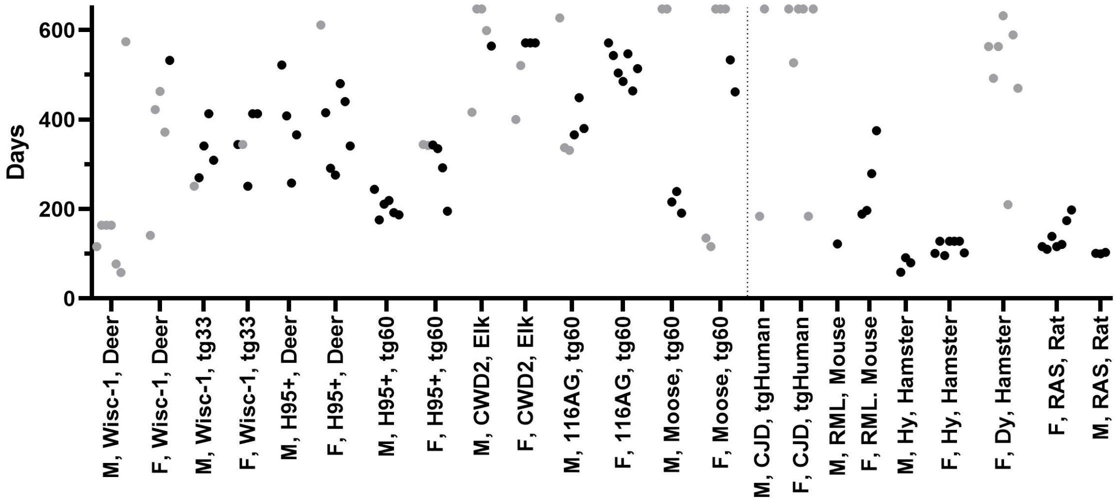

Figure S4. Dot plots of incubation periods in tgBeaver mice. The sex and strain used for the challenge is indicated on the x-axis. Black dots indicate mice with protease resistant PrP in the brain as indicated by western blot and/or immunohistochemical detection. Grey dots are mice that did not develop prion disease.

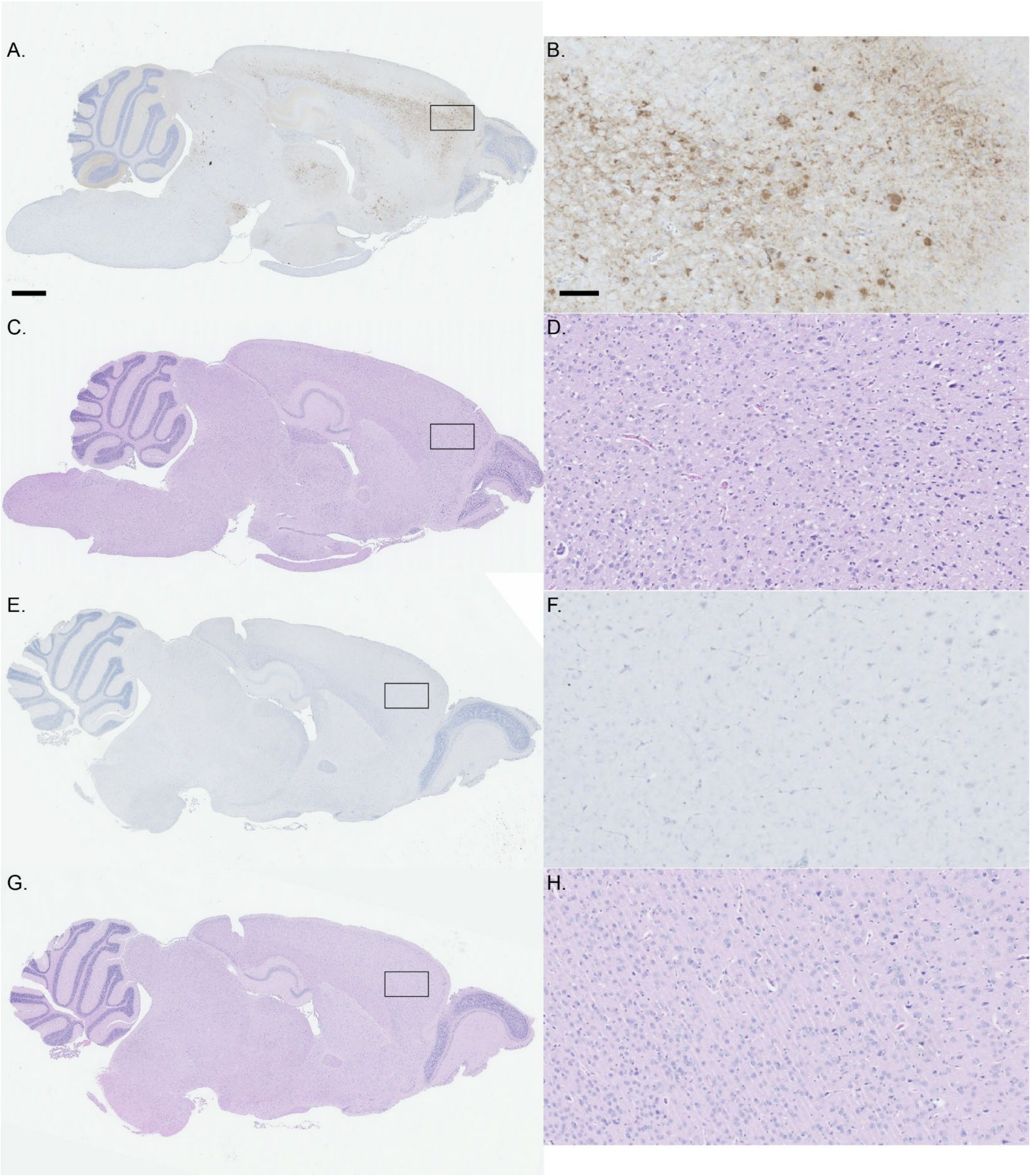

Figure S5. Specificity of immunohistochemical staining in tgBeaver mice. A-D. Neuropathology from a sick tgBeaver mouse inoculated with H95+ prions. E-H. Neuropathology from a healthy, unaffected tgBeaver mouse inoculated with tg60 moose prions collected at the end of the study (647 days). Panels A. B. E. and F were immunostained for PrP using mAb Bar224 1:1,000. C. D. G. and H. were stained with hematoxylin and eosin. A. C. E. and F., the scale bar is 1 mm. B. D. F. and G., the scale bar is 100 um. Panels B. D. F. and H. are magnified images from the panels to their left, (A. C. E. and G.) respectively.
